# The Impact of Herbal Drug Use on Adverse Drug Reaction Profiles of Patients on Antiretroviral Therapy in Zimbabwe

**DOI:** 10.1155/2012/434171

**Published:** 2012-03-19

**Authors:** Tinashe Mudzviti, Charles C. Maponga, Star Khoza, Qing Ma, Gene D. Morse

**Affiliations:** ^1^School of Pharmacy, University of Zimbabwe, P.O. Box MP167, Mount Pleasant, Harare, Zimbabwe; ^2^School of Pharmacy and Pharmaceutical Sciences and the Center of Excellence in Bioinformatics and Life Sciences, University at Buffalo, SUNY, 701 Ellicott Street, Buffalo, NY 14203, USA; ^3^Department of Clinical Pharmacology, University of Zimbabwe, College of Health Sciences, P.O. Box A178, Avondale, Harare, Zimbabwe

## Abstract

*Background*. The main objective was to determine the impact of herbal drug use on adverse drug reactions in patients on antiretroviral therapy (ART). *Methodology*. Patients receiving first-line ART from the national roll-out program participated in this cross-sectional study. Participants were interviewed and a data collection sheet was used to collect information from the corresponding medical record. *Results*. The majority (98.2%) of participants were using at least one herbal drug together with ART. The most common herbal remedies used were *Allium Sativum* (72.7%), *Bidens pilosa* (66.0%), *Eucalyptus globulus* (52.3%), *Moringa oleifera* (44.1%), *Lippia javanica* (36.3%), and *Peltoforum africanum* (34.3%). Two indigenous herbs, *Musakavakadzi* (OR = 0.25; 95% CI 0.076–0.828) and *Peltoforum africanum* (OR = 0.495; 95% CI 0.292–0.839) reduced the occurrence of adverse drug events. *Conclusions*. The use of herbal drugs is high in the HIV-infected population and there is need for pharmacovigilance programs to recognize the role they play in altering ADR profiles.

## 1. Introduction

Several challenges exist in resource-limited settings between balancing the cost and toxicity that occurs during antiretroviral therapy (ART). Most HIV-infected patients in resource-limited settings receive a first-line triple combination of lamivudine, nevirapine, and stavudine or zidovudine [1]. Typical examples of ART in these settings include the World Health Organization prequalified fixed-dose combinations of stavudine/lamivudine/nevirapine (D4T/3TC/NVP) and zidovudine/lamivudine/nevirapine (AZT/3TC/NVP), which are being widely promoted in highly active antiretroviral therapy (HAART) “scale-up” programs.

ART is associated with a variety of adverse drug reactions (ADRs) that can hamper treatment adherence. Particularly, there is concern about the risk for peripheral neuropathy with use of stavudine, especially among patients with lower CD4 cell counts [2] and the risk of rash (including Stevens Johnson syndrome), hypersensitivity, and life-threatening hepatotoxicity with use of nevirapine, especially among women and those with higher CD4 cell counts at initiation of therapy [3–5]. In Sub-Saharan Africa, different incidence rates have been identified for ADRs associated with ART. The most commonly evaluated regimen in Sub-Saharan Africa is the D4T/3TC/NVP combination and this has been associated with various rates of ADRs in different settings [6–13].

Traditional herbal remedies have been used to treat many ailments in Zimbabwe for many years before the introduction of orthodox medicines. The advent of HIV/AIDS in Zimbabwe increased the popularity and use of herbal remedies because antiretroviral drugs were not available at that time [14]. Studies in South Africa have shown that herbal remedies are good supplements to antiretroviral therapy because of their immune boosting properties [15]. A study in western Uganda found that 38% of HIV-positive patients used traditional medicines and antiretroviral drugs at the same time for the management of HIV infection [16]. The major reasons for use of traditional medicines were perceived additional efficacy, improvement in quality of life, and a feeling of control over the disease. The majority of traditional medicines currently being used by patients have not been thoroughly researched. The impact of coadministration of traditional herbal medicine with orthodox medicine has not yet been fully evaluated and consequently there is a lack of data which pertains to this subject. Patients are currently taking herbal medicines with orthodox medicines without the knowledge of how this affects them.

 Research is required to determine factors which affect occurrence of ADRs associated with ART. Identification of patients at different levels of risk may identify subgroups requiring different monitoring intensities. An evaluation of the potential of the most widely used herbal remedies to interact with antiretroviral drugs may be helpful in improving the clinical outcome of patients on ART. It would enable risk identification and assessment, and if necessary, execution of risk reduction strategies. In view of the above, the objective of this study was to determine the impact of coadministration of herbal therapy with ART on ADRs.

## 2. Materials and Methods

### 2.1. Study Site and Population

The study was carried out at the Family Care Centre (FCC) ART clinic in Harare, Zimbabwe. The FCC is part of Parirenyatwa Hospital, which is a major public referral and teaching medical facility. The FCC is integrated into the outpatient department where patients receive antiretroviral drugs and treatments for opportunistic infections for free. The results reported are for patients who had been on first-line or alternate-first-line ART for at least 24 weeks. Inclusion criteria were a documented HIV infection in patients aged 18 years and older on first-line or alternate-first-line HAART for at least 24 weeks. THM use was defined as a daily use of any plant-based extract for a period of at least 24 weeks.

### 2.2. Antiretroviral Drug Treatment

The first line treatment available through the government roll-out programme consisted of a triple fixed dose combination of nevirapine 200 mg, stavudine 30 mg, and lamivudine 150 mg twice daily. Alternate-first-line therapy was available for patients who did not tolerate stavudine and consisted of nevirapine 200 mg, zidovudine 300 mg, and lamivudine 150 mg twice daily. For patients concomitantly taking antituberculosis therapy, efavirenz (EFV) 600 mg once daily at night was prescribed instead of nevirapine as per the national guidelines. ADRs were documented after commencement of ARVs.

### 2.3. Data Collection and Statistical Analysis

Eligible patients gave written consent before being enrolled into the study. The FCC pharmacist and peer counselor, who were not members of the regular ART clinic staff, conducted patient interviews. Patient data that was collected included age, gender, ethnicity, type of ART regimen, comorbidities, and herbal medicine use. Interviews took place in either the local language or English, depending on the participant's preference. Data on ADRs was extracted from the ART patient files. Potential herbal formulations that could influence ADRs were identified using multivariate linear regression and logistic regression analysis. An *a prioiri* significance level of *α* = 0.05 was used for analyses. A logistic regression model was used to test whether herbal therapy use affected the occurrence of ADRs. Three regression models, 1 multiple linear regression model and 2 logistic regression models, were run to identify predictors of ADRs. Data analysis was carried out using the Statistical Analysis System (SAS Version 9.2, Cary, North Carolina, USA).

## 3. Results

### 3.1. Patient Characteristics

A total of 388 patients were interviewed, and of these, 381 were found to be taking at least 1 herbal drug.  Table 1 shows the demographic and clinical characteristics of the study patients. The average age of the patients was 40.8 ± 9.2 years and the majority were female (64.7%). The majority (85.1%) of the patients were on the twice daily regimen of a fixed-dose combination of 30 mg of D4T, 150 mg of 3TC, 200 mg of NVP. The most common comorbidities were malaria (40.0%), tuberculosis (33.8%), shingles (32.2%), and hypertension (17.3%).

### 3.2. Herbal Drug Use

Table 2 shows the frequency of use of herbal remedies. The majority of patients (98.2%) were on at least one indigenous herbal remedy together with their ART regimen. The most common herbal remedies were *Allium Sativum* (72.7%), *Bidens pilosa* (66.0%), *Eucalyptus globulus *(52.3%), *Moringa oleifera* (44.1%), *Lippia javanica* (36.3%), and *Peltoforum africanum* (34.3%). The results from the logistic regression yielded that there was no association between the comorbidities and the severity of the adverse drug reactions. The overall multiple regression model for analyzing the data and interpreting the results was significant for this data analysis (Wald's *P* value = 0.0055).

A one-way ANOVA test revealed a statistically significant difference in the average number of herbs each patient was using and the type of ARV regimen (*F* = 6.40; df = 3,384; *P* = 0.0003). A post hoc analysis revealed that patients on AZT/3TC/EFV were using fewer herbs (mean = 4.0) compared to those using other regimens.

Logistic regression was used to identify potential predictors of ADRs, controlling for other variables. There was a statistically significant association between two herbal medicines and the occurrence of ADRs. Patients who used the indigenous herb, *Musakavakadzi,* were 75 percent less likely to develop ADRs compared to patients who did not use the herb (OR = 0.25; 95% CI: 0.076–0.828; *P* = 0.023). Similarly, patients who used *Peltoforum africanum* were less likely to develop adverse drug events compared to patients who did not use the herb (OR = 0.495; 95% CI: 0.292–0.839; *P* = 0.0091).

### 3.3. Prevalence of Adverse Drug Reactions

Table 3 shows the frequency of ADRs reported by patients. A large proportion of patients experienced at least one ADR during ART treatment (70.4%). The most common ADRs were peripheral neuropathy (41.8%) and skin rash (26.0%). Peripheral neuropathy was mainly reported by patients on regimens that included D4T (93.0%). Skin rash was mainly observed with the NVP-based regimens (88.0%). The other reported ADRs were lipodystrophy, abdominal pain and gastrointestinal symptoms (nausea, vomiting, or heartburn).

## 4. Discussion

The impact of herbal medicine use on the ADR profiles of the first-line regimens used in Zimbabwe was examined. One of the reasons that patients use herbals is to try and alleviate the discomforts caused by antiretroviral ADRs. A relationship was observed between the type of antiretroviral regimen received and the total number of herbs used. Those patients who were on the regimen containing AZT/3TC/EFV used fewer herbal therapies when compared to the other regimens. One possible reason for this could be the decreased rates of ADRs that are associated with this regimen which decreases the need for herbal use for managing toxicity. Another important finding was the relationship that *Musakavakadzi* and *Peltoforum africanum* had with the rates of ADRs. These herbal remedies were associated with decreased prevalence of ADRs, indicating that these herbals might offer some protection to the patients. It is important to note that 98.2 percent of patients were taking at least one herbal remedy and with such a high rate of herbal use there is a need to increase research in herbal remedies that have the potential to influence treatment outcomes.

This study observed a high prevalence (70.2%) of ADRs in patients receiving ART. Of note is the high prevalence of peripheral neuropathy (41.8%) and skin rashes (26.0%). Skin rash occurrences due to ART were comparable to those that were observed in other studies [6–10]. Even though the rates in Zimbabwe are within other reported rates, the prevalence of skin rash is relatively higher. Lipodystrophy rates were lower when compared to studies that were conducted elsewhere [8, 9, 11]. Similarly, GI symptoms and abdominal pain rates were comparable to those found in other studies conducted elsewhere [6, 8, 10].

In light of this high rate of concomitant use of herbal therapy and ART, there is a potential of altered ART pharmacokinetics. Some herbal formulations have been identified as having pharmacokinetic interactions with ARVs. Leaf extracts of *Moringa oleifera *have been established as having *in vitro* CYP3A4 inhibitory activity [17]. Extracts of other plants such as Sutherlandia and grapefruit juice have been found to also inhibit CYP3A4 [18]. ART in a resource-limited setting where herbal use is high needs to make provision for intensifying the monitoring of herbal medicine use and the potential effects it can have on therapy [19].

Identification of specific herbal medicines that influence ADRs forms a key step in developing further studies that will aim to explore the mechanism of action through which they elicit their action. Unfortunately, because of the limited laboratory support, the study could only record ADRs that were clinically apparent. Future studies need to be carried out that will identify more factors predisposing HIV-positive patients to toxicities caused by ART.

## 5. Conclusion

The study observed a high prevalence of concomitant use of herbal medicines with ART. There was a correlation between 2 herbal preparations (*Musakavakadzi* and *Peltoforum africanum*) and a low incidence of ADRs. There is a need to develop pharmacovigilance programs that accommodate herbal medicines as factors that influence ADR prevalence.

## Figures and Tables

**Table 1:** Demographic and clinical characteristics of study patients. Unless specified, figures represent frequencies and percentages.

Characteristic	*N*	%
Age (mean ± SD)	40.8	9.2
Gender		
Male	137	35.3
Female	251	64.7
Comorbidities		
Diabetes mellitus	5	1.3
Hypertension	67	17.3
Anaemia	34	8.8
Asthma	21	5.4
Epilespy	4	1.0
Malaria	155	40.0
Tuberculosis	131	33.8
Shingles	125	32.2
ARV regimen		
AZT/3TC/EFV	10	2.6
AZT/3TC/NVP	36	9.3
D4T/3TC/EFV	12	3.1
D4T/3TC/NVP	330	85.1
Number of herbs per patient (mean ± SD)	7.9	4.4

**Table 2:** Frequencies of herbal use in study population.

Characteristic	*N*	%
*Hypoxis hemerocallidea*	41	10.6
*Dicoma anomala*	103	26.6
*Aloe vera*	108	27.8
*Moringa oleifera*	171	44.1
*Murunguyane* ^†^	62	16.0
*Musakavakadzi* ^†^	13	3.4
*Musosote* ^†^	15	3.9
*Bidens pilosa*	256	66.0
*Lippia javanica*	141	36.3
*Peltoforum africanum*	133	34.3
*Ngoka 11* ^†^	30	7.7
*Symphytum officinale*	41	10.6
*Eucalyptus globules*	203	52.3
*Allium Sativum*	282	72.7

^†^Scientific names could not be identified for these formulations

**Table 3:** Prevalence of adverse drug reactions in the study patients.

ADR type and severity	*N*	%
ADR prevalence	273	70.4
ADR severity (*N* = 273)		
Grade 1	161	41.5
Grade 2	96	24.7
Grade 3	15	3.9
Grade 4	1	0.3
Type of ADRs		
Peripheral neuropathy	162	41.8
Skin rash	101	26.0
Lipodystrophy	13	3.4
GI symptoms	28	7.2
